# Combined local and systemic immunization is essential for durable T-cell mediated heterosubtypic immunity against influenza A virus

**DOI:** 10.1038/srep20137

**Published:** 2016-02-01

**Authors:** Ida E. M. Uddback, Line M. I. Pedersen, Sara R. Pedersen, Maria A. Steffensen, Peter J. Holst, Allan R. Thomsen, Jan P. Christensen

**Affiliations:** 1 Department of Immunology and Microbiology, University of Copenhagen, Copenhagen, Denmark

## Abstract

The threat from unpredictable influenza virus pandemics necessitates the development of a new type of influenza vaccine. Since the internal proteins are highly conserved, induction of T cells targeting these antigens may provide the solution. Indeed, adenoviral (Ad) vectors expressing flu nucleoprotein have previously been found to induce short-term protection in mice. In this study we confirm that systemic (subcutaneous (s.c.) immunization rapidly induced heterosubtypic protection predominantly mediated by CD8 T cells, but within three months clinical protection completely disappeared. Local (intranasal (i.n.)) immunization elicited delayed, but more lasting protection despite relatively inefficient immunization. However, by far, the most robust protection was induced by simultaneous, combined (i.n. + s.c.) vaccination, and, notably, in this case clinical protection lasted at least 8 months without showing any evidence of fading. Interestingly, the superior ability of the latter group to resist reinfection correlated with a higher number of antigen-specific CD8 T cells in the spleen. Thus, detailed analysis of the underlying CD8 T cell responses highlights the importance of T cells already positioned in the lungs prior to challenge, but at the same time underscores an important back-up role for circulating antigen-specific cells with the capacity to expand and infiltrate the infected lungs.

Recurrent influenza virus epidemics represent an important constant health threat to modern society. Annual epidemics cause about 250,000–500,000 deaths per year worldwide (http:/www.who.int/mediacentre/factsheet/fs211/en/), and occasional pandemic strains may kill several million people within the first 12 months of their circulation. Compared to seasonal influenza, pandemics are associated with a higher proportion of severely affected children, young adults and pregnant women. Thus, development of highly efficient influenza vaccines is a very important public health issue.

Several types of influenza vaccines are available for use against the seasonal epidemics, including inactivated vaccines and live attenuated virus^1^. The protection afforded by inactivated influenza vaccines is predominantly antibody mediated, targeting the major surface antigens, hemagglutinin and, to some extent, neuraminidase^1^. These antigens are subject to gradual mutational changes over time (antigenic drift); nevertheless, in most years it is possible based on a global surveillance network, to predict the most likely antigenic patterns for the upcoming winter season and prepare relevant vaccines in advance. This is unlike the situation for pandemic strains, which are typically the result of stochastic genetic reassortment (antigenic shift) in birds and swine co-infected with different influenza strains carrying surface antigens that are new to the human population. Therefore, most individuals will be susceptible to infection and if these new viruses are easily transmitted between humans, a pandemic will be the result. Because a pattern regarding the changes in surface antigens cannot be predicted in these cases, conventional vaccine strategies fail to provide protective vaccines relevant in the early phases of a pandemic. Consequently, vaccinologists are searching for other ways to induce rapid protection against severe influenza infection.

Cold adapted, attenuated live vaccines could represent an answer in this quest, since these viruses induce not only a humoral response, but, additionally, a cellular immune response targeting the much more conserved structural and non-structural internal antigens^1^,^2^. However, these vaccines are not recommended in infants, elderly, or immune-compromised individuals because of their potential to induce pathogenic reactions. Furthermore, the cold-adapted vaccine approach has serious limitations when it comes to the development of vaccines for avian zoonotic flu strains, as these do not replicate in the human upper respiratory tract where the appropriate temperatures are found^3^,^4^. For these reasons other approaches are being tested aiming to elicit broad cross-protective immunity towards a variety of influenza A strains. One possible approach is targeting conserved elements on the surface molecule hemagglutinin^1^,^5^. Thus the stalk of the hemagglutinin represents an interesting vaccine target with up to 85% identity between different subtypes and about 95% identity within the same subtype. So far it has been found that certain experimentally generated monoclonal antibodies may be broadly neutralizing^6^, but no vaccine has been developed that will readily induce these types of antibodies in the general population^7^. Another potential target is the M2 surface antigen, which is relatively conserved amongst many different influenza A virus strains^1^,^8^. This antigen is not highly expressed on the virion itself^9^, but represents a valid target for a humoral immune response on influenza infected host cells^10^,^11^ that express this antigen in abundance^12^. Finally, virus vectored vaccines targeting the much more conserved internal antigens are also being pursued^13^,^14^,^15^,^16^,^17^,^18^. Both animal experiments and studies in humans clearly indicate that cross-protective T-cell mediated immunity may significantly modify the outcome of influenza virus infection^2^,^19^,^20^,^21^,^22^,^23^,^24^ and if such cells could be deliberately primed and expanded, it would be expected that severe disease could be avoided.

Adenoviral (Ad) vectors offer many advantages when it comes to the induction of cell-mediated immunity. They are safe, easy to produce in large amounts and can be applied by several routes including mucosal application. Perhaps most importantly, they have been found to be superior to other viral vectors including pox vectors when it comes to rapid induction of strong CD8 T-cell responses^25^,^26^,^27^. CD8 T cells have traditionally been assigned the major role in cell-mediated immunity to influenza^19^,^23^, although recently CD4 T cells have received renewed attention as an important effector subset in the control of this infection^24^,^28^,^29^. Immunization with viral vectors expressing NP have quite a long history and Ad vectors expressing NP has been found to transiently protect mice^30^,^31^,^32^,^33^,^34^,^35^. Notably, virtually all existing studies to evaluate protection against viral challenge have been performed no later than a few months after vaccination, and our studies clearly demonstrate that an evaluation performed this early after vaccination substantially overestimates the induced protection. Thus to our knowledge no study exclusively involving a (T-cell inducing) NP targeting vaccine has been found to demonstrate substantial protection beyond a couple of months. This is in contrast to HA targeting Ad vectors that may also induce protection in the long-term^36^,^37^,^38^, but, of course, suffers from the same limitations in breadth as the inactivated vaccine.

In the present study we have reevaluated the potential of using a replication-deficient, adenoviral vector expressing the NP antigen for induction of heterosubtypic protection. Our findings confirm that a single subcutaneous (s.c.) vaccination of naïve individuals with this vector induce protection within few days against different serologically unrelated influenza viruses. However, substantial protection only lasts for two to three months. Mucosal (intranasal (i.n.)) immunization prolongs the period of protection, but by far the most efficient and stable protection is induced by simultaneous immunization by both routes, underscoring the importance of combining local mucosal immunity with potent systemic priming for robust, long-standing (~8 month) protection. We believe this information carries important implications for the design of future vaccine strategies aimed against pandemic flu.

## Results

### Ad5 encoding influenza NP induces a protective CD8 T-cell response

First, to ascertain the immunogenicity of our construct, B6 mice were inoculated s.c. in a hind foot pad, and twelve days later splenocytes were harvested and analyzed by intracellular cytokine staining (ICS) and flow cytometry to determine numbers of antigen-specific effector CD8 T cells producing IFN-γ in response to stimulation with NP peptides. As expected the strongest response was found against the NP366 epitope, which is known to represent the most dominant H-2^b^ restricted influenza NP epitope^39^, while a limited, but still clearly detectable response was measured against the subdominant NP217 epitope. Hardly any response was observed against other epitopes tested (NP17, NP55 and NP97) (data not shown). Notably, a high proportion of the vector generated CD8 T cells co-produced TNF-α (data not shown), indicating that they were fully differentiated antiviral effector cells^40^.

**Figure 1:**
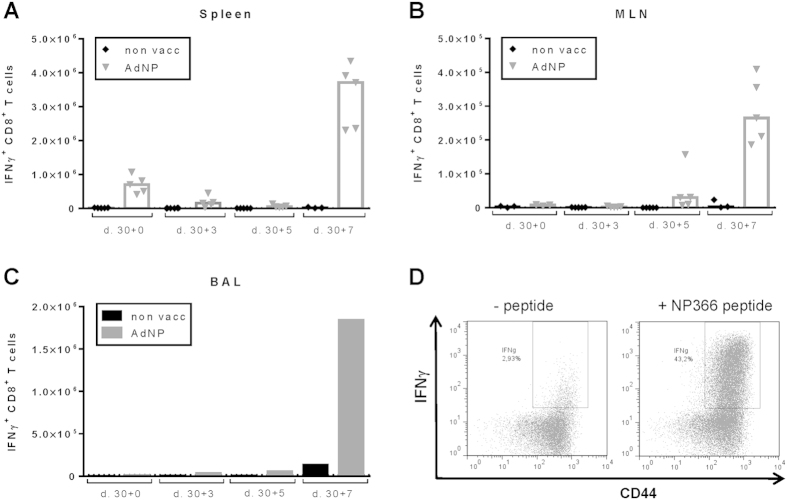
Influenza-specific CD8 T-cell recall in AdNP vaccinated mice. C57BL/6 mice were vaccinated subcutaneously with 2 × 10^7^ PFU of AdNP and 30 days later challenged with PR8 virus. (**A–C**) Spleens (**A**), mediastinal lymph nodes (MLN, **B**) and brochealveolar lavage (BAL, **C**) were harvested before and 3, 5 and 7 days post infection. Antigen-specific CD8 T cells were enumerated using intracellular cytokine staining for IFNγ. For spleens and mediastinal lymph nodes (MLN), each dot represents one animal and bars represent medians. For BAL, bars represent average numbers of cells per mouse based on analysis of a pool from 3–5 mice. (**D**) Representative dot plots for gated CD8 T cells from BAL harvested 7 days after virus challenge and incubated in the absence or presence of NP366 peptide. Please note that for cells harvested from an inflammatory site, significant numbers of cytokine-producing cells will be found even without *ex vivo* stimulation; we consider these cells to represent *in vivo* activated influenza-specific CD8 T cells.

**Figure 2:**
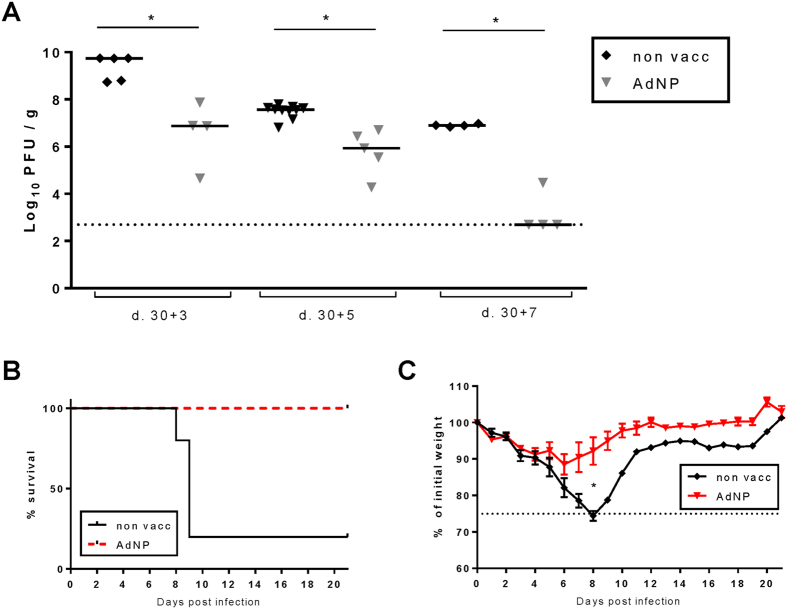
Conferred protection from immunization with AdNP. C57BL/6 mice were vaccinated subcutaneously with 2 × 10^7^ PFU of AdNP and challenged with PR8 virus intranasally 30 days later. (**A**) Lungs were removed 3, 5 and 7 days post infection and viral titers were analysed using an MDCK plaque assay. Each dot represents one animal and lines represent medians. Dotted line represents the detection level for the assay. (**B–C**) Line graphs show survival (**B**) and percentages of initial body weight (**C**) as a function of time after challenge (5 animals per group). *p < 0.05.

To determine if vaccination affected the course of subsequent infection, B6 mice vaccinated 30 days before together with unvaccinated controls were challenged i.n. with a lethal dose of PR8. Viral titers in the lungs and the accompanying T-cell response were determined before challenge and 3, 5, and 7 days later (Figs 1A–D and 2A). Parallel groups of mice were followed to study the clinical severity of influenza virus infection (Fig. 2B,C). It is evident from this analysis that vaccinated mice generated an accelerated recall response demonstrable in all three anatomical compartments, but it is also noted that, although virus-specific CD8 T cells can be found earlier, a substantial CD8 T-cell influx into the lungs could not be observed until about 7 days after challenge (Fig. 1A–C). However, despite the low numbers of CD8 T cells present in the lungs at earlier time points, significantly lower viral loads were detected in the lungs on all days of analysis (Fig. 2A), and, perhaps most importantly, nearly all unvaccinated mice died from the challenge, whereas no vaccinated mice became severely ill or died (Fig. 2B,C). These results confirmed the potential of the vaccine to induce relevant protection against influenza virus infection.

### AdNP induces heterosubtypic immunity

**Figure 3:**
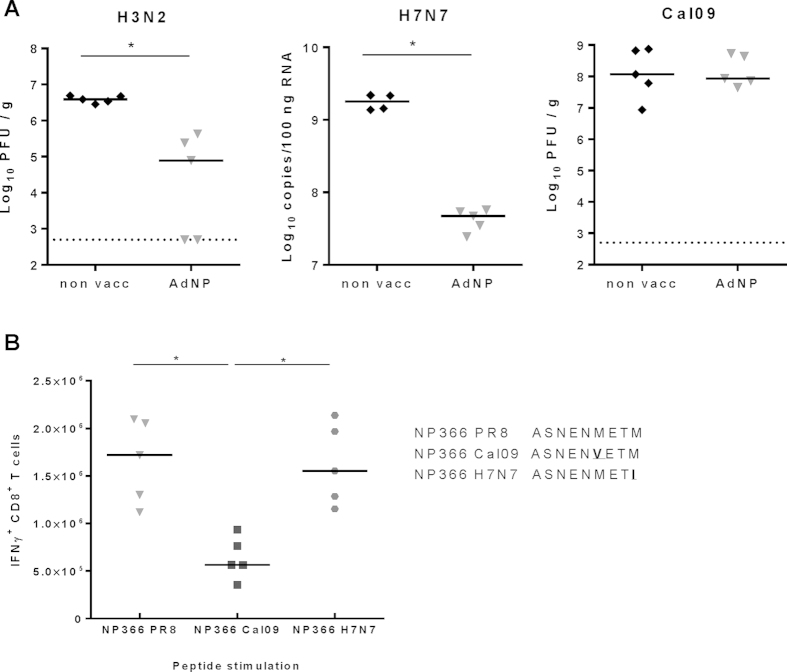
AdNP induces hetorosubtypic protection. (**A**) C57BL/6 mice were vaccinated subcutaneously with 2 × 10^7^ PFU of AdNP and 30 days later challenged with H3N2 virus, H7N7 virus or Cal09 virus intranasally. Lungs were isolated 5 days post challenge and viral titers were measured using either an MDCK plaque assay (H3N2 and Cal09) or RT-qPCR (H7N7). Dotted line represents the detection level for the MDCK plaque assay. (**B**) Evaluation of cross-reactivity of NP366-specific CD8 T cells from AdNP vaccinated mice against NP366 epitopes from H7N7 and Cal09. No. of IFNγ+ CD8 T cells detected following stimulation with NP366 peptides from the indicated virus stains. Stimulation with NP366 from PR8 is the positive control as the AdNP epitope originates from this strain. Epitope sequences are shown with mutations in bold. Each dot represents one animal and lines represent group medians. *p < 0.05.

To confirm the breadth of the vaccine induced protection, vaccinated mice and unvaccinated controls were challenged i.n. with 2 serologically distant influenza A virus strains, and 5 days later the animals were sacrificed and lung virus burden was determined either by plaque titration or by qRT-PCR. As can be seen in Fig. 3A, prior vaccination resulted in significantly lower viral loads both in the case of an H3N2 strain and an H7N7 strain indicating that the vaccine was able to induce a broad protection against serologically unrelated influenza strains.

Regarding the basis for viral control, cell depletion studies confirmed a key role for CD8 T cells (data not shown). We could even pin-point the specificity of the major CD8 T-cell subset by challenging vaccinated mice with H1N1 Cal09. This virus strain differs in the amino acid sequence of the major H-2^b^ restricted NP366 epitope from that expressed by the vaccine. This difference markedly impacts the capacity of vaccine primed CD8 T cells to react with target cells expressing the variant peptide (Fig. 3B). Thus, while CD8 T cells from mice vaccinated with NP of PR8 were able to cross-react strongly with the variant epitope expressed in the H7N7 strain, much fewer of these cells were able to recognize the epitope expressed by Cal09, and corresponding to this, no reduction of lung virus titers was observed in vaccinated mice challenged with this virus strain (Fig. 3A).

### Peripheral AdNP vaccination causes rapid protection, which fades over time

**Figure 4:**
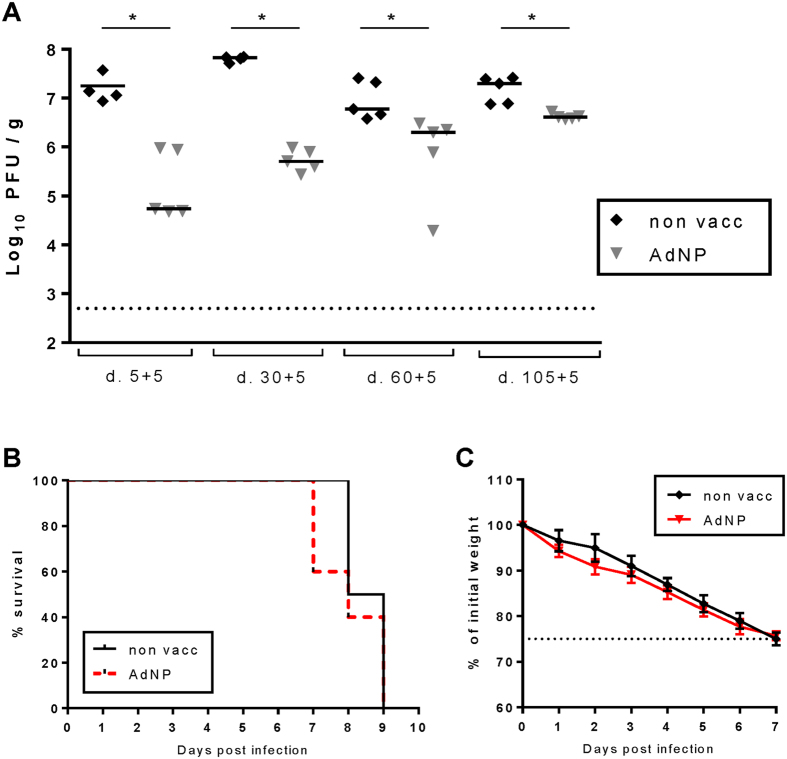
Vaccine-induced protection as a function of time after vaccination. C57BL/6 mice were vaccinated subcutaneously with 2 × 10^7^ PFU of AdNP and 5, 30, 60 or 105 days later challenged with PR8 virus. (**A**) Five days post challenge, lungs were isolated and viral titers were determined using an MDCK plaque assay. Each dot represents one mouse and lines represent medians. Dotted line represents detection level for the assay. (**B**–**C**) Line graphs showing survival (**B**) and weight loss (**C**) of vaccinated mice challenged with PR8 virus 120 days later. Animals were monitored daily and euthanized if the weight loss exceeded 25%. The dots represent the means and bars SEMs. The dotted line represents the humane end point (25% weight loss). *p < 0.05

A key issue for a vaccine is the ability to induce long-standing protection. To address the longevity of vector-induced protection, mice vaccinated 5, 30, 60 or 105 days earlier were challenged with a standard dose of homologous virus and 5 days later the mice were sacrificed and the viral titers in the lungs were determined. As can be seen in Fig. 4, vaccinated mice were already significantly protected 5 days after vaccination; this protection was antigen specific, since vaccination with an irrelevant vector did not induce any protection (data not shown), and about the same level of protection was maintained for at least 1 month. However, between 30 and 105 days after vaccination antiviral protection faded gradually, although in most experiments lung virus titers were still significantly lower in challenged mice vaccinated 3–4 month earlier. Importantly, the small reduction in lung virus titers observed in mice challenged 3–4 month p.v. was not reflected in clinical parameters such as morbidity and mortality. Thus, the virus-induced weight loss and mortality of mice vaccinated 3–4 month earlier in most experiments were similar to that of unvaccinated mice (Fig. 4B,C).

### On the importance of T-cell distribution

**Figure 5:**
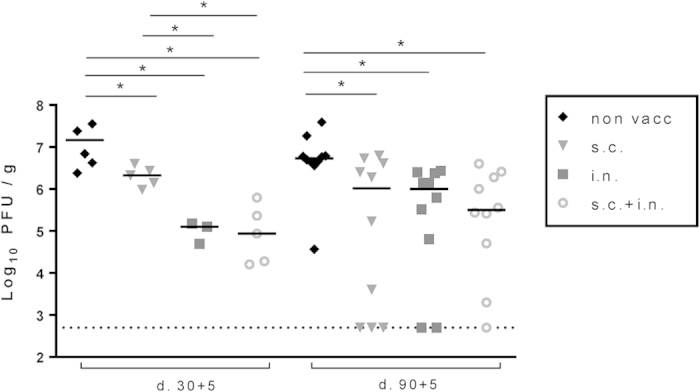
Vaccination induced protection as a function of time and route of immunization. C57BL/6 mice were vaccinated either subcutaneously (s.c.), intranasally (i.n.) or via both routes (i.n.+s.c.) with 2 × 10^7^ PFU of AdNP. Thirty and 90 days later 5 animals from each group and unvaccinated controls were challenged with PR8 virus i.n. and lungs were isolated 5 days later. Viral titer in lungs was determined using an MDCK plaque assay. Each dot represents one animal and lines represent group medians. Dotted line represents detection level for the assay. *p < 0.05.

Previous results have stressed the importance of T cells in the lung airways as an important first line of defence against influenza virus infection of the lower respiratory tract^22^, and mucosal vaccination targeting NP has been found to afford protection early (3 weeks) after vaccination^31^. In an attempt to mimic naturally induced influenza immunity, we decided to modify our vaccine strategy and vaccinate mice either i.n. or i.n. combined with s.c. administration at the same time. The capacity of these mice to control an influenza virus challenge was studied and compared to their level of systemic and local CD8 T-cell memory at the time of virus challenge. Mice were challenged with PR8 either 30 or 90 days after vaccination, and viral loads were determined 5 days after challenge (Fig. 5). Initially (d. 30), virus titers were lower in both groups of mice subjected to local (i.n.) immunization compared to systemic (s.c.) immunization alone; however, with time the capacity to protect tended to decrease in i.n. vaccinated mice, while protection seemed more stable in the mice vaccinated both s.c. and i.n.

**Figure 6:**
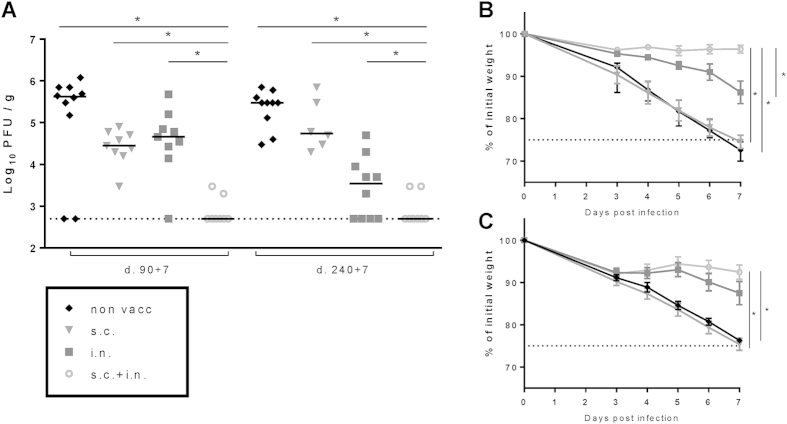
*In vivo* protection as a function of route of immunization. C57BL/6 mice were vaccinated either subcutanoursly (s.c.), intranasally (i.n.) or via both routes (i.n.+s.c.) and 90 or 240 days later challenged with PR8 virus i.n. (**A**) Seven days post challenge animals were sacrificed and lung viral titers were determined by an MDCK plaque assay. Each dot represents one animal and bars represent the group medians. The dotted line represents the detection level of the assay. (**B**–**C**) Weight loss measured up until 7 days post infection (**B**) mice infected 90 days earlier; (**C)** mice infected 240 days earlier). Dots represent means and bars SEM. Dotted line represents the humane end-point (>25% weight loss). *p < 0.05.

Assuming that an appearance of a difference between i.n. and i.n.+s.c. vaccinated mice regarding their capacity to control the infection reflected that recruitment of additional effector cells from the circulation became more important with time after vaccination, we decided to evaluate the difference in viral loads 7 days after infection, expecting that the contribution of recruited cells would play an even bigger role with increased time after challenge (for validating of this prediction, see below). Indeed, the superiority of combined vaccination became clear when we evaluated viral loads 7 days after challenge of mice vaccinated 90 or 240 days earlier (Fig. 6A). Now we found that mice previously vaccinated by both routes had significantly less virus remaining in their lungs compared to mice vaccinated only i.n. or s.c.

Additionally, when we looked at the infection induced weight loss, s.c. vaccinated mice did not differ from unvaccinated mice, whereas mice vaccinated i.n. or i.n. plus s.c. were both initially protected (Fig. 6B,C). However, protection was more durable in the mice given combined vaccination, albeit the difference did not reach the level of statistical significance for the mice vaccinated 240 days earlier. Please, note, the degree of antiviral protection did not show any evidence of decreasing between day 90 and 240 p.v., indicating that a very stable state of immunity is induced in these mice.

**Figure 7:**
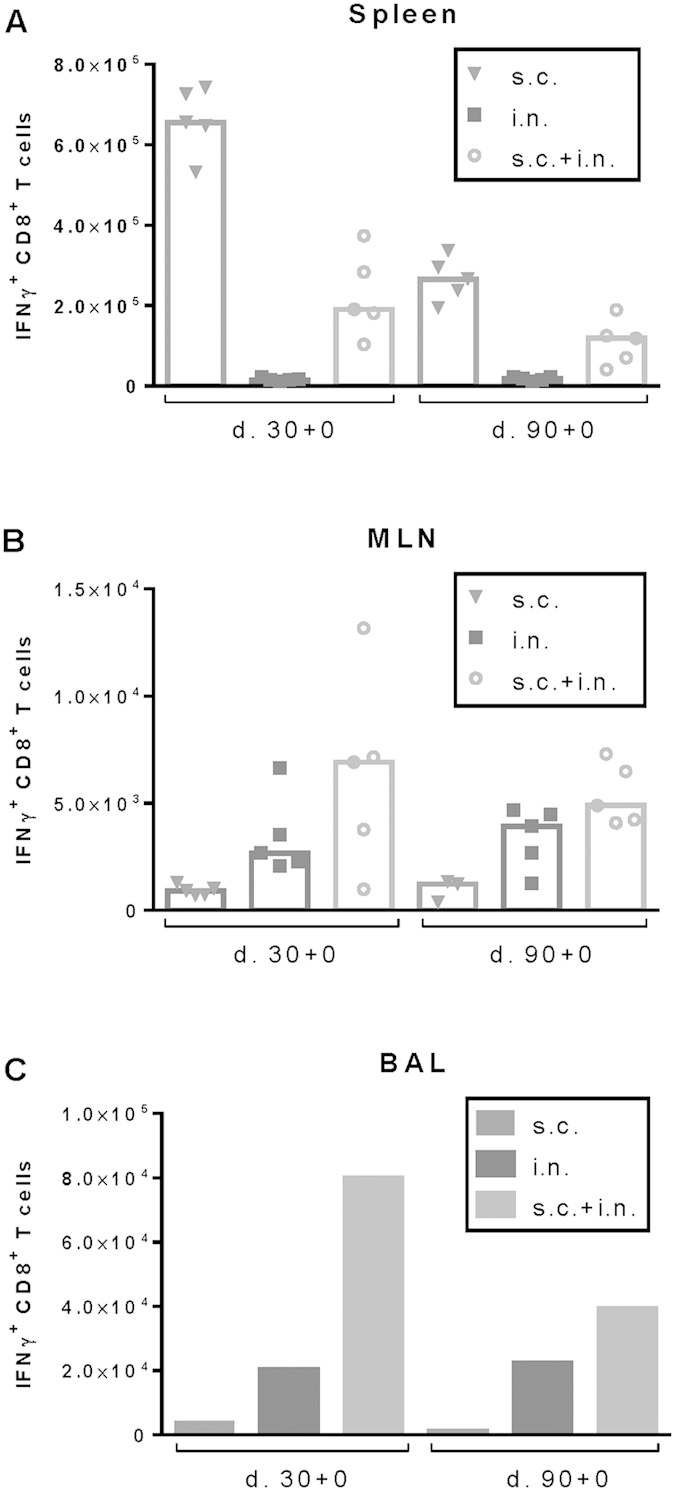
Distribution of NP366-specific CD8 T cells as a function of route of immunization. C57BL/6 mice were vaccinated either subcutaneously (s.c.), intranasally (i.n.) or via both routes (i.n.+s.c.) with 2 × 10^7^ PFU of AdNP. Thirty and 90 days later animals were sacrificed and spleens (**A**), MLNs (**B**) and BAL (**C**) were isolated. No. of IFNγ+ CD8+ T cells in each organ site were determined though flow analysis after peptide stimulation. Each dot represents one animal and bars represent medians (spleen and MLN). In BAL bars represent average no of IFNγ+ CD8+ T cells per animal based on a pool from 5 animals.

**Figure 8:**
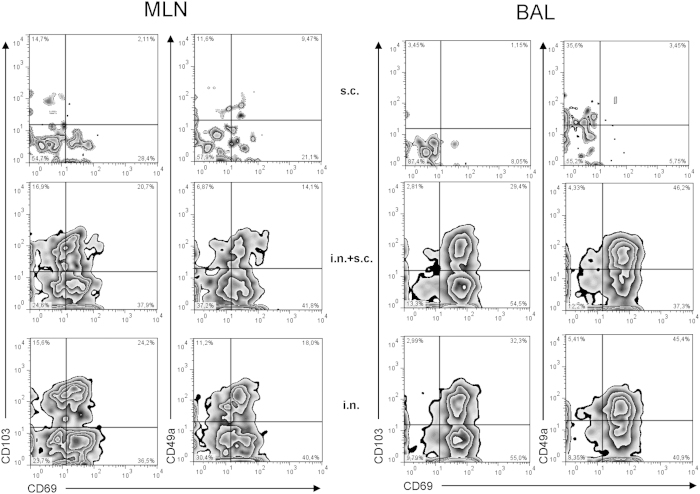
Phenotype of vaccine-induced memory CD8 T cells in MLNs and BAL is impacted by the route of immunization. C57BL/6 mice were vaccinated either subcutaneously (s.c.), intranasally (i.n.) or via both routes (i.n.+s.c.) with 2 × 10^7^ PFU of AdNP Thirty days later animals were sacrificed and MLNs and BAL were harvested. Tetramer^+^ CD8^+^ T cells in from each organ site were analyzed for the expression of CD69, CD103 and CD49a. Contour plots representative of 4 mice/group are depicted. The experiment was performed on two separate occasions with similar results.

Studies into the distribution of antiviral CD8 T cells prior to challenge (Fig. 7), revealed that early protection reflected the presence of a preexisting population of influenza-specific CD8 T cells in BAL to a higher degree than the number of virus-specific cells in the circulating pool, as evaluated by analysis of splenocytes. Furthermore, 30 days after i.n. priming either alone or combined with systemic immunization, a substantial subset of CD69^+^ memory CD8 T cells in the MLNs co-expressed CD49a and CD103 as did NP-specific CD8 T cells recovered from the BAL (Fig. 8). Since high expression of CD49a and CD103 has been linked to retention in the lung and extravasation through mucosal epithelium, respectively^41^,^42^, these findings point to a propensity of NP-specific CD8 T cells in these mice to interact with residual antigen in the MLNs and receive signals for localization in the lungs^43^.

**Figure 9:**
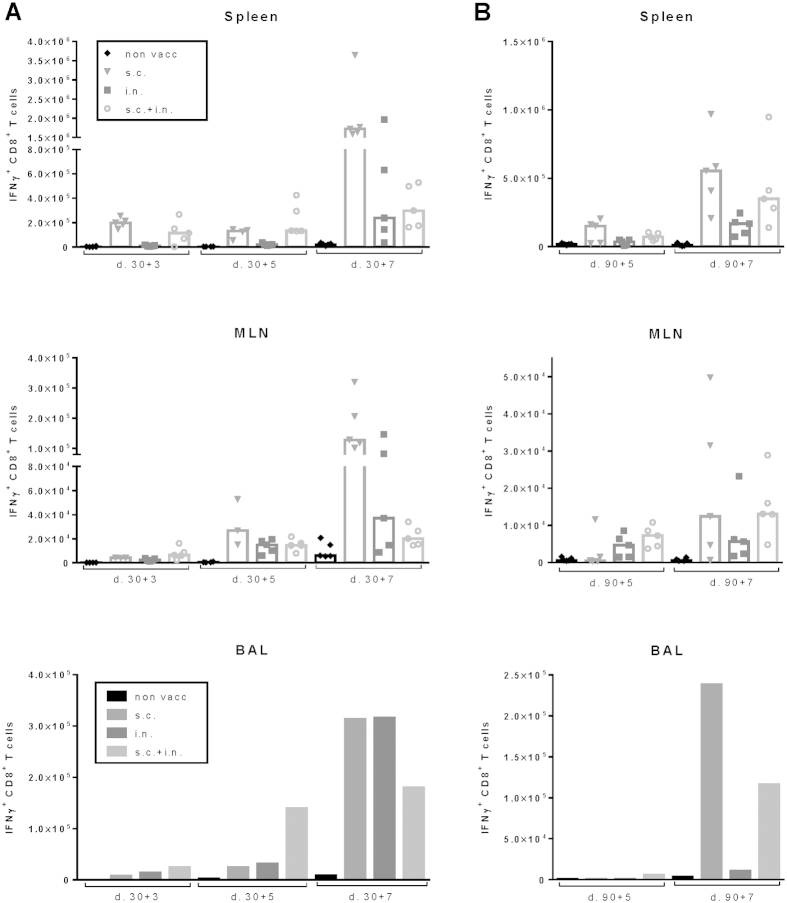
Distribution of NP366-specific CD8 T cells following challenge of mice vaccinated by different routes. C57BL/6 mice were vaccinated either subcutaneously (s.c.), intranasally (i.n.) or via both routes (i.n.+s.c.) with 2 × 10^7^ PFU of AdNP 30 days (**A**) and 90 days (**B**) later animals were challenged i.n. with PR8 virus. Three, 5 and 7 days post challenge animals were sacrificed and spleens, MLNs and BAL were isolated. No. of IFNγ+ CD8 T cells in each organ site were determined through flow analysis after peptide stimulation. Each dot represents one animal and bars represent medians (spleen and MLN). In BAL, bars represent average no of IFNγ+ CD8+ T cells per animal based on a pool from 5 animals.

As expected from the data on virus control (Fig. 6), additional analyses of the CD8 T cell response following viral challenge revealed slightly different kinetics whether studied early (d.30) or late (d.90) after vaccination (Fig. 9). Thus, early after vaccination combined local and systemic vaccination was associated with a pronounced rapid T-cell infiltration of the lungs that seemingly made a more prolonged response redundant (Fig. 9A). In contrast, at 90 days post vaccination a sustained influx was observed in both groups of mice that were vaccinated s.c. (Fig. 9B). Importantly, unlike the situation in s.c. vaccinated mice, low grade T-cell infiltration was already evident by d. 5 post challenge in those mice that had also received an i.n. vaccination. Taken together these findings suggested that combined vaccination would lead to the most efficient CD8 T-cell response, combining early protection exerted by local cells with the capacity for a more sustained T-cell influx when needed.

### Local immunity critically depends on CD8 effector T cells

**Figure 10:**
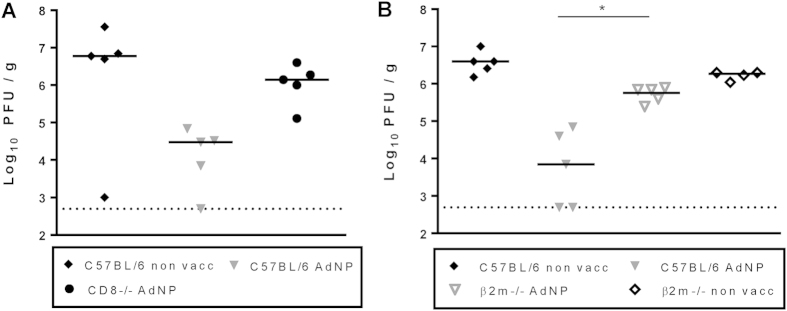
Role of CD8 T cells in vaccine induced protection following combined vaccination. C57BL/6, CD8−/− (**A**) and β2m−/− (**B**) mice were vaccinated s.c. as well as i.n. with 2 × 10^7^ PFU of AdNP and 30 days later challenged with PR8 virus. Five days after challenge animals were sacrificed and lung viral titers were determined using an MDCK plaque assay. Each dot represents one animal and the lines denote group medians. Dotted line represents detection level of MDCK plaque assay. *p < 0.05.

Since local vaccination potentially could invoke immunological effector systems not effectively activated through parental immunization, we found it pertinent to reaffirm the key role of CD8 effector T cells under these conditions. Because we did not expect Abs to effectively deplete T cells already localized in the lung airways, we used genetically modified (β2m−/− or CD8−/−) mice for this analysis. These mice together with matched WT mice were vaccinated i.n. plus s.c. , and 30 days later, both vaccinated mice and unvaccinated controls were challenged i.n. with PR8; 5 days later the viral burden in the lungs was determined. As can be seen in Fig. 10, only vaccinated WT mice, but not β2m−/− or CD8−/− mice were substantially protected against influenza challenge. From this, we conclude that CD8 T cells represent the major antiviral effector arm also in mice undergoing combined vaccination.

### Combined vaccination induces protection early after vaccination

**Figure 11:**
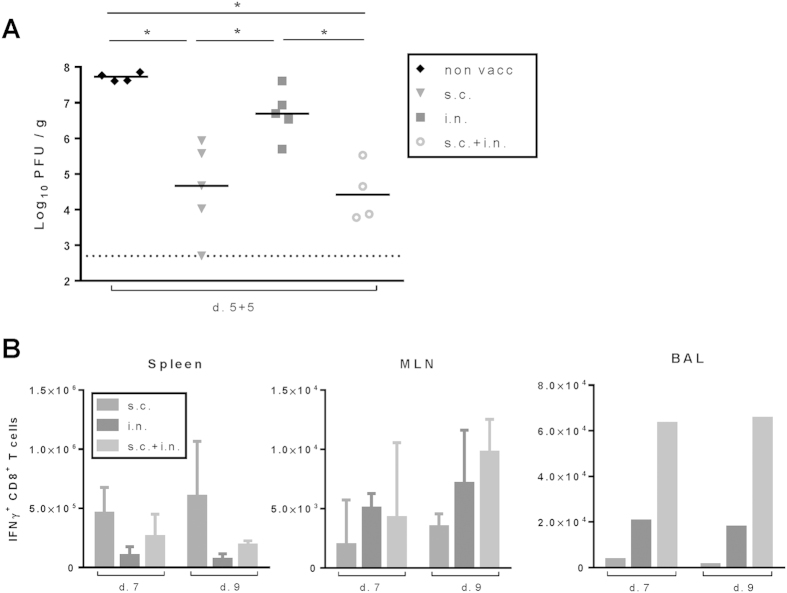
Rapid protection induced by AdNP vaccination depends on rote of immunization. C57BL/6 mice were vaccinated either subcutaneously (s.c.), intranasally (i.n.) or via both routes (i.n.+s.c.) with 2 × 10^7^ PFU of AdNP. (**A**) Five days after vaccination animals were challenged with PR8 virus intranasally and 5 days later animals were sacrificed and lungs were isolated. Viral titers were determined using an MDCK plaque assay. Each dot represents one animal and the lines denote group medians. Dotted line represents detection level for the assay. (**B**) Seven and 9 days after vaccination, similarly vaccinated animals were sacrificed and spleens, MLNs and BAL were isolated. No. of IFNγ+ CD8 T cells were determined through flow analysis after peptide stimulation. Medians and ranges of 5 animals are depicted for spleen and MLNs. In BAL bars represent average no of IFNγ+ CD8 T cells per animal based on a pool from 5 animals.*p < 0.05

Finally, based on the finding that numbers of circulating virus-specific CD8 T cells were reduced in mice subjected to combined immunization compared to mice vaccinated only s.c. (Fig. 7A), we decided to test if the combined vaccination would impede the induction of clinical protection. This could represent a critical issue for a vaccine, which would be relevant to apply under the threat of an emerging pandemic. To study this, groups of WT mice were vaccinated i.n., s.c. or through both routes, and 5 days later these mice and unvaccinated control were challenged i.n. with PR8. As can be seen in Fig. 11A , mice vaccinated s.c. whether alone or in combination with i.n. vaccination were well protected against subsequent influenza infection, while a lesser degree of protection was observed in the mice vaccinated only i.n.

To understand the background for this pattern, we followed the early phase of the CD8 T-cell response elicited in mice vaccinated i.n., s.c. or by both routes (Fig. 11B). While combined vaccination may appear to impair the accumulation of virus-specific CD8 T cells in the circulating pool compared to mice undergoing only s.c. immunization, this primarily seems to reflect a redistribution of the generated effector T cells to the lungs, as also indicated by our previous analysis of memory cell distribution in the two situations. The high number of circulating effector cells rapidly generated in both groups of systemically vaccinated mice readily explains why these routes of immunization are associated with protection early after vaccination; this is unlike the situation following i.n. immunization, which seems to represent a rather inefficient way of inducing a strong (systemic) immune response.

## Discussion

Influenza A virus is a very successful virus and an important health problem of modern society. Split or subunit vaccines represent the most common influenza A vaccines currently in use, and these predominantly target the surface antigen hemagglutinin, which is subject to continuous change either through mutations or reassortment with non-human virus variants. Consequential to a vaccine production time for conventional flu vaccines of about ½ year, the efficiency of vaccines targeting this antigen is limited by our ability to predict the antigenic make-up of the virus strain(s) circulating in the human population about 6 month into the future. With regard to influenza variants with a potential to start a pandemic, we cannot make predictions due to the stochastic nature of the underlying reassortment, and it is therefore pertinent to think outside the box to design vaccines inducing clinical protection to future pandemic strains. As mentioned earlier, the internal antigens of influenza A virus are much more conserved than hemagglutinin and neuraminidase^44^,^2^. However, for a vaccine against the former antigens to provide protection, it is required that a cell-mediated immune response be elicited and, importantly, sustained. Viral vectors are good at inducing cell-mediated immunity, and non-replicating adenovirus encoding the antigen of interest is among the best vector systems in this context^25^,^26^,^27^. In this report we have reevaluated the vaccine potential of an adenovirus vaccine targeting the NP of influenza A. Our results confirm that a single systemic (s.c.) vaccination in influenza A-naïve animals suffices for generation of a strong CD8 T-cell response. Additionally, we show that this response protects the mice from lethal i.n. flu challenge as early as 5 days p.v. Regarding the breadth of vaccine induced protection, our results reveal clear heterosubtypic protection.

With respect to the specificity of the involved T cells, compelling evidence for a key role of CD8 T cells targeting NP366 was obtained using Cal09 virus that has a single amino acid substitution in the normally dominant NP366 epitope compared to PR8 and thus also our vaccine. Consistent with published findings^45^,^46^ head-to-head comparison of the activity of vaccine induced T cells against the wild type epitope and the mutated peptide revealed a marked reduction in the ability of wild type primed CD8 T cells to recognize the variant sequence, and no *in vivo* protection was observed.

Obviously this result also exposes that viral escape in the major T-cell targeted epitope(s) represents a potential weakness of our vaccine strategy, as previously pointed out in murine studies on flu-induced cross-protection^46^,^47^. However, first of all, unlike inbred mice, the majority of humans expresses a set of 6 potential MHC class I molecules for antigen presentation, not just 2–3, so the risk of a complete failure to recognize viral NP variants is much lower. Secondly, the experimental set-up chosen for these studies is deliberately made simple - targeting only a single viral gene – in order to increase the transparency of the results. For actual vaccination of humans we foresee a vaccine cocktail targeting multiple conserved antigens so that complete viral escape also for this reason becomes highly unlikely.

While systemic vaccination alone can protect the mice, protective capacity rapidly fades. At about 3 months p.v. the capacity to control influenza virus replication is reduced to such a degree that even though a significant decrease in lung virus burden compared to unvaccinated controls may still be found, this is not sufficient to prevent challenged mice from becoming severely ill. Gradual fading of T-cell mediated clinical protection as a function of time has been observed before in cases where an immediate T-cell response is imperative^2^,^22^,^48^,^49^,^50^. Besides representing a gradual decline in the number of airway associated T cells^22^,^51^, this very likely also reflect an intrinsic decline in CD8 T-cell functionality with time after last stimulation^52^,^53^,^54^. Detailed studies on T-cell based immunity to secondary flu infection have revealed several important lines of defence to the invading virus^22^. Thus local T cells in the airways represent a critical first line of defence, while recruitment of circulating effector memory cells make up a second line, and the progeny of restimulated central memory cells are the ultimate mediators involved in clearing a secondary infection. Based on these convincing studies we decided to test if we could extend the period of protection by priming i.n. and thus starting with a higher number of T cells programmed for lung migration^43^. Indeed, our results suggested that the decline in local protection could be delayed by immunizing the mice in this manner. Overall this route of immunization is not very efficient, however, and fewer circulating CD8 T cells were generated. This was reflected in delayed induction of protective immunity and fewer NP-specific cells detected in the spleen. Consequently, based on the model described above, it was feared that the second and third lines of defence would be less efficient in mice vaccinated exclusively through this route. Therefore, inspired by studies conducted with the purpose of inducing immunity against pulmonary tuberculosis^55^,^56^, we included in our experiments mice that were vaccinated at the same time both i.n. and s.c. Results from these mice exceeded our expectations. Combined vaccination led to the same rapid induction of protection also observed in s.c. immunized mice. This is clearly relevant in a pandemic situation, where rapid protection of vaccinees may be the key to early containment. More importantly, compared to i.n. vaccination, combined vaccination seemed to afford the mice with more robust and much prolonged immune protection in the lungs; to our knowledge such stable protection has not been seen before in the context of what essentially represent a single immunization approach. Clearly the presence of influenza specific CD8 T cells in the lungs prior to challenge is important and represent a good predictor for an early reduction of virus replication in the lungs. However, as pointed out in pioneering work from the group of D. Woodland^22^, recruitment of circulating cells also matters in the long run. Our results clearly document that the accumulation of primed cells takes place more efficiently in mice subjected to combined vaccination than in i.n. vaccinated mice, in which the pool of circulating antigen-specific CD8 T cells is smaller.

Looking at the practical implications of this study, we find it demonstrated that simultaneous i.n. and s.c. vaccination represents a superior vaccine approach to immunize for respiratory viral infections compared to immunization by either route alone. Furthermore, both immunizations can be delivered at the same time simplifying logistics compared to prime-boost regimens that are often required for prolonged protection. Clearly, when it comes to human vaccination, Ad5 needs to be replaced by another Ad vector because of the high level of antivector immunity directed towards this vector. However, several non-human Ad vectors matching Ad5 in both safety and immunogenicity have been described^57^,^58^. Also, should it prove very difficult to achieve approval by regulatory agencies to apply an adenobased vaccine i.n., combining s.c. adenovector immunization with already licensed live, attenuated influenza virus might be a viable alternative – with the limitations already mentioned.

How do our observations affect a pan-influenza vaccine strategy based on T-cell mediated immunity? The idea driving the development of a broadly protective influenza A vaccine is to have a vaccine that will induce protection in those situations where no pre-existing conventional antibody-based vaccine will work. Even though an antibody based vaccine will always provide earlier and better protection, this type of vaccine currently requires a good match between vaccine antigen and the targeted H and N molecules, and this is exactly what will lack in the early phases of an emerging pandemic. In light of this, there are basically two alternatives for a vaccine directed towards influenza pandemics: One is to have a T-cell based vaccine targeting conserved internal antigens stock-piled and ready to be handed out in a critical situation. Under these conditions the poor longevity of protection induced by systemic vaccination represents a lesser problem. However, this approach has two limitations. First, it is pertinent that the vaccine is bought and paid for even before we realistically foresee a pandemic, otherwise no manufacturer is likely to produce it. This is a political issue. The other limitation is that the vaccine needs to induce protection rapidly following immunization. From our study it seems that both s.c. and combined s.c. and i.n. immunization fulfills this requirement. As an alternative to having the vaccine in stock, one could imagine having the general population vaccinated with a cross protective vaccine even before there is an immediate pandemic threat. In this case the longevity of T-cell mediated protection becomes a critical issue, and the present results strongly suggest that combined local and systemic vaccination may represent a way to address this problem.

## Materials and Methods

### Mice

Female C57BL/6 (WT B6) mice, 6-8 weeks old, were obtained from Taconic Farms (Ry, Denmark) and housed in a specific pathogen–free facility. Also beta-2-microglobulin deficient (β2m −/−) mice and CD8 deficient mice, both on a C57BL/6 background, were obtained from Taconic Farms. Perforin deficient and IFN-γ deficient mice were originally obtained from The Jackson Laboratory, and IFN-γ/perforin double-deficient mice were generated locally through intercrossing of these strains, as previously described^59^. Upon arrival, all mice were allowed to acclimatize for ≥1 wk at the facility before being used in experiments. All experimental procedures were approved by the national animal ethics committee (The Animal Experiments Inspectorate) and were conducted in accordance with national Danish guidelines; the mice were housed in an AAALAC accredited facility in accordance with good animal practice as defined by FELASA.

### Adenoviral vectors

A replication-deficient E1-deleted Ad serotype 5 vector with a nonfunctional E3 gene, expressing the NP from influenza strain A/Puerto Rico/8/34 (designated AdNP) was produced as described previously^60^. Adenoviral particles were purified using standard methods, aliquoted, and frozen at −80°C in 10% glycerol. Insert was verified by sequencing (data not shown). Infectivity of the adenovirus stocks was determined using Adeno-X Rapid Titer Kit (Clontech Laboratories, Mountain View, CA).

### Vaccination

Vaccination was given s.c. and/or i.n. In the case of s.c. vaccination, mice were briefly anesthetized with isoflourane and injected in the right foot pad with 2 × 10^7^ particle forming units (PFU) in 30 μl of PBS. Mice vaccinated i.n. were first anaesthetized by intraperitoneal (i.p.) injection with avertin (2,2,2 tribromoethanol in 2-methyl-2-butanol, 250 mg/kg) and then vaccinated with 2 × 10^7^ PFU in 30 μl of PBS in the nostrils.

### *In vivo* depletion of CD4 and CD8 T cells

100 μg α‐CD4 monoclonal antibodies (mAb) (YTS 191.1.2 and YTA 3.1.2) and/or α‐CD8 mAb (YTS 156.7.7 and YTS 169.4.2.1) were administered i.p. day −1 and +1 relative to influenza challenge. All hybridomas were kind gifts from S Cobbold, University of Oxford, UK^61^,^62^. Cell depletion was verified by flow cytometric analysis.

### Virus challenge

The influenza viruses listed in Table 1 were used for challenge studies. For each virus preparation, the lethal dose was determined, and 1-3 LD_50_ were used for challenge, except in the case of X31, which do not induce lethal disease in WT B6 mice. Mice to be challenged were first anaesthetized by i.p. injection with avertin (2,2,2 tribromoethanol in 2-methyl-2-butanol, 250 mg/kg), and subsequently infected i.n. with 30 μl of appropriately diluted influenza virus. After influenza infection, mice were followed by daily weights and euthanized by cervical dislocation if the weight loss exceeded 25%; alternatively the experiment was terminated 21 days post challenge.

### Influenza virus plaque assay

Lungs were homogenized using sterilized sand and a mortar and pestle, 1% FBS in PBS was added to obtain a 10% weight/volume suspension. Samples were spun down at 600 G for 15 min at 4 °C, and the supernatant was transferred to new tube and kept on ice until use.

MDCK cells were used for influenza plaque assay and grown in complete medium. 4.5 × 10^4^ MDCK cells in 100 μl medium were grown in 96-well plates overnight. For the plaque assay, 10-fold dilutions of the lung suspensions were prepared using an influenza growth medium containing DMEM 1965 medium with 2 mM L-glutamin, 200 IU/ml penicillin, 50 μg/ml streptomycin, 0.2% BSA, 1% sodium-pyruvate and 5 units/ml TPCK Trypsin. MDCK-cells were first washed twice with PBS and then incubated with 50 μl of virus dilution for 2 hour at 37 °C, 5% CO2. Samples were then removed, and an overlay medium containing 2× minimum essential medium (MEM) eagle supplemented with 0.4% BSA, 10% NaHCO3, 2% Streptomycin, 2% penicillin and 5 units/ml TPCK trypsin mixed 1:1 with 1.8% methylcellulose was added to the cells. Cells were incubated for 48 hour at 37°C, 5% CO2. Then overlay was removed and wells were washed 2x with PBS. Cells were fixated with 4% formaldehyde in PBS for 30 min, RT. After fixation cells were washed twice with PBS and permeabilized with warm 0.5% Triton-X in Hanks balanced salt solution medium for 10 min, RT. Cells were subsequently washed twice with PBS. Next, cells were incubated with primary α-influenza nucleocapsid A mAb (Nordic Biosite) diluted 1:1500 in 1% BSA in PBS for 1 hour at 37°C, 5% CO2. Antibody was removed and cells washed 5x. This was followed by incubation with a secondary goat α-mouse HRP conjugated mAb (Dako) diluted 1:500 in 1% BSA in PBS for 1 hour at 37 °C, 5% CO2. After secondary antibody incubation cells were washed 5x with PBS. 200 μl substrate solution containing 3 mg/ml 3-amino-9-ethylcarbazole and 0.07% H_2_O_2_ and 5 mM citrate phosphate buffer pH5 was added to wells for 30 minutes, RT. Substrate was removed and cells were washed once with PBS before counting. All samples were run in duplicates. Plaque forming units per g lung tissue were calculated accordingly:


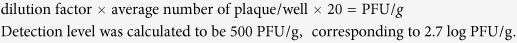


### Quantitative real time polymerase chain reaction (qRT-PCR)

In some experiments, viral loads were determined using qRT-PCR. The influenza M gene was absolutely quantified while the housekeeping gene, murine glyceraldehyde-3-phosphate dehydrogenase (mGAPDH), was included in the amplifications to address inter-PCR variations. The reliability of this assay was initially validated through correlation with infective titers as determined by plaque assay.

For the analysis the Brilliant II QRT-PCR, 1-step kit (Agilent Technologies) was used. The following was added to each well: 100 ng purified RNA, 12.5 μl master mix, 0.5 μl of each primer (10 μM), 0.5 μl mGAPDH probe (10 μM), 0.75 μl M probe (10 μM), 0.375 μl (1:500) reference dye and 1 μl RT/RNase block enzyme mixture diluted in Milli-Q water to a total volume of 25 μl. See table 2 for primers and probes used.

Samples were run on an Mx3000P Real-Time QPCR instrument (Agilent Technologies) according to the following settings; 30 min/50 °C, 10 min/95 °C followed by 40 cycles of 30 sec/95 °C, 1 min/58 °C and 30 sec/72 °C. All samples were run in triplicates and standard curves in duplicates.

### Preparation of single-cell suspensions and flow cytometry

To obtain single cells from spleens and mediastinal lymph node (MLN), these organs were aseptically removed and pressed through a fine steel mesh (70 μm). For brochoalveolar lavage (BAL) sampling, avertin anaesthetized mice were first exsanguinated in order to reduce the risk of lymphocyte contamination from the blood. Then the trachea was exposed and a small incision was made. A venflon was inserted into the incision and the lungs were flushed 3 times with ice-cold Hanks BSS medium. BAL samples were pooled within groups in order to obtain enough cells for analysis. All samples were centrifuged and resuspended in Hanks BSS. Cells were then counted on an automated cell counter, Countess (Invitrogen). This was followed by centrifugation and resuspension in RPMI 1640 cell culture medium containing 10% FCS supplemented with 2-ME, l-glutamine, and penicillin-streptomycin. For enumeration of Ag-specific T cells, splenocytes were incubated at 37°C and 5% CO_2_ for 5 h in the presence of 1 μg/ml relevant peptide, 50 IU/ml IL-2, and 3 μM monensin. Cells were then stained for surface markers. Subsequent to surface staining, the cells were washed, permeabilized, and stained for intracellular cytokines^63^. Samples were analyzed using a LSR II (BD Biosciences), and data analysis was conducted using FlowJo software (TreeStar) according to the gating strategy depicted in suppl. Figure 1. NP366-specific CD8 T cells from mediastinal LNs and BAL were phenotyped using a combination of NP366-tetramers, anti-CD8, anti-CD69, anti-CD49a and anti-CD103.

### Antibodies for flow cytometry

The following fluorochrome‐conjugated monoclonal rat anti-mouse antibodies were used for surface and intracellular cytokine staining: PerCP-Cy5.5 conjugated α-CD8a (clone 53–6.7), FITC conjugated α‐CD4 (clone GK1.5), Pacific blue conjugated α‐B220 (clone RA3-6B2), APC‐Cy7 conjugated α‐CD44 (clone IM7), APC conjugated α‐IFNγ (clone XMG1.2), PECy7 conjugated α –TNFα (clone MP6, XT22), FITC conjugated α-CD69 (clone H1.2F3), PE conjugated α-CD49a (clone HMα1) and BV421 conjugated α-CD103 (clone 2E7). All antibodies were purchased from Biolegend. Relevant tetramers were kindly provided by Søren Buus, this institute.

### Statistical analysis

Analysis was performed using Graphpad Prism software v6.01. Statistical comparison of two groups was done using the Mann-Whitney test (two-tailed). Data from all experiments with more than two groups were first compared using a one-way ANOVA test, and, only if groups were found to differ significantly, this analysis was followed by pair-wise comparisons using Mann-Whitney rank sum test. Statistical significant differences between two groups were acknowledged if p <0.05; statistical significance is marked with an asterix (*).

## Additional Information

**How to cite this article**: Uddback, I. E. M. *et al*. Combined local and systemic immunization is essential for durable T-cell mediated heterosubtypic immunity against influenza A virus. *Sci. Rep*. **6**, 20137; doi: 10.1038/srep20137 (2016).

## Supplementary Material

Supplementary Information

## Figures and Tables

**Table 1:** Influenza strains used in the study.

Virus strain	Serotype	Dose in 30 μl PBS	Dose in PFU	Refered to as
A/Puerto Rico/8/34	H1N1	1–3 LD50	100 PFU	PR8
A/California/07/2009	H1N1	1–3 LD50	9,9 × 10^3^ PFU	Cal09
A/Hong Kong/X31	H3N2	<1 LD50	3.4 × 10^4^ PFU	X31
A/Equine/London/1416/73	H7N7	1–3 LD50		H7N7

**Table 2:** Primers and Probes used for qRT-PCR.

Primers and probes	Sequence (5′–3′)
M forward primer	AGA TGA GTC TTC TAA CCG AGG TCG
M reverse primer	TGC AAA AAC ATC TTC AAG TCT CTG
M probe	FAM-TCA GGC CCC CTC AAA GCC GA-BHQ-1
mGAPDH forward primer	CAA TGT GTC CGT CGT GGA
mGAPDH reverse primer	GAT GCC TGC TTC ACC ACC
mGAPDH probe	HEX-CGC CTG GAG AAA CCT GCC AAG TAT-BHQ-1

Primers and probes were synthesized at TAQ Copenhagen A/S. FAM: Carboxyfluorescein, BHQ-1: BlackHoleQuencher-1, HEX: Hexachlorofluorescein.

## References

[b1] Wong S. S. & Webby R. J. Traditional and new influenza vaccines. Clin. Microbiol. Rev. 26, 476–492 (2013). 23824369 10.1128/CMR.00097-12 PMC3719499

[b2] Powell T. J. . Priming with cold-adapted influenza A does not prevent infection but elicits long-lived protection against supralethal challenge with heterosubtypic virus. J. Immunol. 178, 1030–1038 (2007). 17202366 10.4049/jimmunol.178.2.1030

[b3] Shinya K. . Avian flu: influenza virus receptors in the human airway. Nature 440, 435–436 (2006). 16554799 10.1038/440435a

[b4] van R. D. . H5N1 Virus Attachment to Lower Respiratory Tract. Science 312, 399 (2006). 16556800 10.1126/science.1125548

[b5] Nabel G. J. & Fauci A. S. Induction of unnatural immunity: prospects for a broadly protective universal influenza vaccine. Nat. Med. 16, 1389–1391 (2010). 21135852 10.1038/nm1210-1389

[b6] Throsby M. . Heterosubtypic neutralizing monoclonal antibodies cross-protective against H5N1 and H1N1 recovered from human IgM+ memory B cells. PLoS. One. 3, e3942 (2008). 19079604 10.1371/journal.pone.0003942 PMC2596486

[b7] Kanekiyo M. . Self-assembling influenza nanoparticle vaccines elicit broadly neutralizing H1N1 antibodies. Nature 499, 102–106 (2013). 23698367 10.1038/nature12202 PMC8312026

[b8] Grandea A. G. III . Human antibodies reveal a protective epitope that is highly conserved among human and nonhuman influenza A viruses. Proc. Natl. Acad. Sci. USA 107, 12658–12663 (2010). 20615945 10.1073/pnas.0911806107 PMC2906546

[b9] Rossman J. S. & Lamb R. A. Influenza virus assembly and budding. Virology 411, 229–236 (2011). 21237476 10.1016/j.virol.2010.12.003 PMC3086653

[b10] Slepushkin V. A. . Protection of mice against influenza A virus challenge by vaccination with baculovirus-expressed M2 protein. Vaccine 13, 1399–1402 (1995). 8578816 10.1016/0264-410x(95)92777-y

[b11] Andersson A. M. . Increased immunogenicity and protective efficacy of influenza M2e fused to a tetramerizing protein. PLoS. One. 7, e46395 (2012). 23049700 10.1371/journal.pone.0046395 PMC3462204

[b12] Zebedee S. L. & Lamb R. A. Influenza A virus M2 protein: monoclonal antibody restriction of virus growth and detection of M2 in virions. J. Virol. 62, 2762–2772 (1988). 2455818 10.1128/jvi.62.8.2762-2772.1988 PMC253710

[b13] Brewoo J. N. . Cross-protective immunity against multiple influenza virus subtypes by a novel modified vaccinia Ankara (MVA) vectored vaccine in mice. Vaccine 31, 1848–1855 (2013). 23376279 10.1016/j.vaccine.2013.01.038 PMC4224110

[b14] Hessel A. . MVA vectors expressing conserved influenza proteins protect mice against lethal challenge with H5N1, H9N2 and H7N1 viruses. PLoS. One. 9, e88340 (2014). 24523886 10.1371/journal.pone.0088340 PMC3921149

[b15] Lillie P. J. . Preliminary assessment of the efficacy of a T-cell-based influenza vaccine, MVA-NP+M1, in humans. Clin. Infect. Dis. 55, 19–25 (2012). 22441650 10.1093/cid/cis327 PMC3369564

[b16] Mbawuike I. N., Zhang Y. & Couch R. B. Control of mucosal virus infection by influenza nucleoprotein-specific CD8^+^ cytotoxic T lymphocytes. Respir. Res. 8, 44 (2007). 17597533 10.1186/1465-9921-8-44 PMC1914056

[b17] Berglund P., Fleeton M. N., Smerdou C. & Liljestrom P. Immunization with recombinant Semliki Forest virus induces protection against influenza challenge in mice. Vaccine 17, 497–507 (1999). 10073729 10.1016/s0264-410x(98)00224-2

[b18] Tripp R. A. & Tompkins S. M. Virus-vectored influenza virus vaccines. Viruses. 6, 3055–3079 (2014). 25105278 10.3390/v6083055 PMC4147686

[b19] Altenburg A. F., Rimmelzwaan G. F. & de Vries R. D. Virus-specific T cells as correlate of (cross-) protective immunity against influenza. Vaccine 33, 500–506 (2015). 25498210 10.1016/j.vaccine.2014.11.054

[b20] Christensen J. P., Doherty P. C., Branum K. C. & Riberdy J. M. Profound protection against respiratory challenge with a lethal H7N7 influenza A virus by increasing the magnitude of CD8(+) T-cell memory. J. Virol. 74, 11690–11696 (2000). 11090168 10.1128/jvi.74.24.11690-11696.2000 PMC112451

[b21] Epstein S. L. Prior H1N1 influenza infection and susceptibility of Cleveland Family Study participants during the H2N2 pandemic of 1957: an experiment of nature. J. Infect. Dis. 193, 49–53 (2006). 16323131 10.1086/498980

[b22] Hikono H. . T-cell memory and recall responses to respiratory virus infections. Immunol. Rev. 211, 119–132 (2006). 16824122 10.1111/j.0105-2896.2006.00385.x

[b23] McMichael A. J., Gotch F. M., Noble G. R. & Beare P. A. Cytotoxic T-cell immunity to influenza. N. Engl. J. Med. 309, 13–17 (1983). 6602294 10.1056/NEJM198307073090103

[b24] Strutt T. M. . Multipronged CD4(+) T-cell effector and memory responses cooperate to provide potent immunity against respiratory virus. Immunol. Rev. 255, 149–164 (2013). 23947353 10.1111/imr.12088 PMC4206082

[b25] Barefoot B. . Comparison of multiple vaccine vectors in a single heterologous prime-boost trial. Vaccine 26, 6108–6118 (2008). 18809447 10.1016/j.vaccine.2008.09.007 PMC2646904

[b26] Bett A. J. . Comparison of T cell immune responses induced by vectored HIV vaccines in non-human primates and humans. Vaccine 28, 7881–7889 (2010). 20937317 10.1016/j.vaccine.2010.09.079

[b27] Shiver J. W. . Replication-incompetent adenoviral vaccine vector elicits effective anti-immunodeficiency-virus immunity. Nature 415, 331–335 (2002). 11797011 10.1038/415331a

[b28] Wilkinson T. M. . Preexisting influenza-specific CD4^+^ T cells correlate with disease protection against influenza challenge in humans. Nat. Med. 18, 274–280 (2012). 22286307 10.1038/nm.2612

[b29] Hogan R. J. . Protection from respiratory virus infections can be mediated by antigen-specific CD4(+) T cells that persist in the lungs. J. Exp. Med. 193, 981–986 (2001). 11304559 10.1084/jem.193.8.981 PMC2193400

[b30] Epstein S. L. . Protection against multiple influenza A subtypes by vaccination with highly conserved nucleoprotein. Vaccine 23, 5404–5410 (2005). 16011865 10.1016/j.vaccine.2005.04.047

[b31] Kim S. H. . Mucosal vaccination with recombinant adenovirus encoding nucleoprotein provides potent protection against influenza virus infection. PLoS. One. 8, e75460 (2013). 24086536 10.1371/journal.pone.0075460 PMC3783479

[b32] Lambe T. . Immunity against heterosubtypic influenza virus induced by adenovirus and MVA expressing nucleoprotein and matrix protein-1. Sci. Rep. 3, 1443 (2013). 23485942 10.1038/srep01443 PMC3595699

[b33] Price G. E. . Single-dose mucosal immunization with a candidate universal influenza vaccine provides rapid protection from virulent H5N1, H3N2 and H1N1 viruses. PLoS. One. 5, e13162 (2010). 20976273 10.1371/journal.pone.0013162 PMC2953831

[b34] Vitelli A. . Vaccination to conserved influenza antigens in mice using a novel Simian adenovirus vector, PanAd3, derived from the bonobo Pan paniscus. PLoS. One. 8, e55435 (2013). 23536756 10.1371/journal.pone.0055435 PMC3594242

[b35] Moraes T. J., Lin G. H., Wen T. & Watts T. H. Incorporation of 4-1BB ligand into an adenovirus vaccine vector increases the number of functional antigen-specific CD8 T cells and enhances the duration of protection against influenza-induced respiratory disease. Vaccine 29, 6301–6312 (2011). 21704101 10.1016/j.vaccine.2011.06.022

[b36] Hoelscher M. A. . New pre-pandemic influenza vaccines: an egg- and adjuvant-independent human adenoviral vector strategy induces long-lasting protective immune responses in mice. Clin. Pharmacol. Ther. 82, 665–671 (2007). 17957181 10.1038/sj.clpt.6100418 PMC2793094

[b37] Kim E. H. . Intranasal adenovirus-vectored vaccine for induction of long-lasting humoral immunity-mediated broad protection against influenza in mice. J. Virol. 88, 9693–9703 (2014). 24920793 10.1128/JVI.00823-14 PMC4136366

[b38] Vemula S. V. . Broadly protective adenovirus-based multivalent vaccines against highly pathogenic avian influenza viruses for pandemic preparedness. PLoS. One. 8, e62496 (2013). 23638099 10.1371/journal.pone.0062496 PMC3640067

[b39] Townsend A. R. . The epitopes of influenza nucleoprotein recognized by cytotoxic T lymphocytes can be defined with short synthetic peptides. Cell 44, 959–968 (1986). 2420472 10.1016/0092-8674(86)90019-x

[b40] Lichterfeld M. . HIV-1-specific cytotoxicity is preferentially mediated by a subset of CD8(+) T cells producing both interferon-gamma and tumor necrosis factor-alpha. Blood 104, 487–494 (2004). 15059848 10.1182/blood-2003-12-4341

[b41] Ray S. J. . The collagen binding alpha1beta1 integrin VLA-1 regulates CD8 T cell-mediated immune protection against heterologous influenza infection. Immunity. 20, 167–179 (2004). 14975239 10.1016/s1074-7613(04)00021-4

[b42] Sung S. S. . A major lung CD103 (alphaE)-beta7 integrin-positive epithelial dendritic cell population expressing Langerin and tight junction proteins. J. Immunol. 176, 2161–2172 (2006). 16455972 10.4049/jimmunol.176.4.2161

[b43] Takamura S. . The route of priming influences the ability of respiratory virus-specific memory CD8^+^ T cells to be activated by residual antigen. J. Exp. Med. 207, 1153–1160 (2010). 20457758 10.1084/jem.20090283 PMC2882830

[b44] Hayashida H., Toh H., Kikuno R. & Miyata T. Evolution of influenza virus genes. Mol. Biol. Evol. 2, 289–303 (1985). 3870863 10.1093/oxfordjournals.molbev.a040352

[b45] Guo H. & Topham D. J. Multiple distinct forms of CD8^+^ T cell cross-reactivity and specificities revealed after 2009 H1N1 influenza A virus infection in mice. PLoS. One. 7, e46166 (2012). 23029425 10.1371/journal.pone.0046166 PMC3459832

[b46] Zhong W. . Significant impact of sequence variations in the nucleoprotein on CD8 T cell-mediated cross-protection against influenza A virus infections. PLoS. One. 5, e10583 (2010). 20485501 10.1371/journal.pone.0010583 PMC2868023

[b47] Sage L. K., Fox J. M., Tompkins S. M. & Tripp R. A. Subsisting H1N1 influenza memory responses are insufficient to protect from pandemic H1N1 influenza challenge in C57BL/6 mice. J. Gen. Virol. 94, 1701–1711 (2013). 23580424 10.1099/vir.0.049494-0 PMC3749524

[b48] Jensen S. . Adenovirus-based vaccine against Listeria monocytogenes: extending the concept of invariant chain linkage. J. Immunol. 191, 4152–4164 (2013). 24043891 10.4049/jimmunol.1301290

[b49] Williams M. A. Instant recall: a key role for effector-phenotype CD8(+) memory T cells in immune protection. Immunity. 38, 1090–1091 (2013). 23809159 10.1016/j.immuni.2013.06.007

[b50] Olson J. A., McDonald-Hyman C., Jameson S. C. & Hamilton S. E. Effector-like CD8(+) T cells in the memory population mediate potent protective immunity. Immunity 38, 1250–1260 (2013). 23746652 10.1016/j.immuni.2013.05.009 PMC3703254

[b51] Hogan R. J. . Activated antigen-specific CD8^+^ T cells persist in the lungs following recovery from respiratory virus infections. J. Immunol. 166, 1813–1822 (2001). 11160228 10.4049/jimmunol.166.3.1813

[b52] Kedzierska K., Valkenburg S. A., Doherty P. C., Davenport M. P. & Venturi V. Use it or lose it: establishment and persistence of T cell memory. Front Immunol. 3, 357 (2012). 23230439 10.3389/fimmu.2012.00357 PMC3515894

[b53] Kundig T. M. . On T cell memory: arguments for antigen dependence. Immunol. Rev. 150, 63–90 (1996). 8782702 10.1111/j.1600-065x.1996.tb00696.x

[b54] Roberts A. D., Ely K. H. & Woodland D. L. Differential contributions of central and effector memory T cells to recall responses. J. Exp. Med. 202, 123–133 (2005). 15983064 10.1084/jem.20050137 PMC2212898

[b55] Santosuosso M., McCormick S., Zhang X., Zganiacz A. & Xing Z. Intranasal boosting with an adenovirus-vectored vaccine markedly enhances protection by parenteral Mycobacterium bovis BCG immunization against pulmonary tuberculosis. Infect. Immun. 74, 4634–4643 (2006). 16861651 10.1128/IAI.00517-06 PMC1539608

[b56] Tchilian E. Z. . Simultaneous immunization against tuberculosis. PLoS. One. 6, e27477 (2011). 22110657 10.1371/journal.pone.0027477 PMC3217972

[b57] Barnes E. . Novel adenovirus-based vaccines induce broad and sustained T cell responses to HCV in man. Sci. Transl. Med. 4, 115ra1 (2012). 10.1126/scitranslmed.3003155 PMC3627207 22218690

[b58] Colloca S. . Vaccine vectors derived from a large collection of simian adenoviruses induce potent cellular immunity across multiple species. Sci. Transl. Med. 4, 115ra2 (2012). 10.1126/scitranslmed.3002925 PMC3627206 22218691

[b59] Nansen A. . Compromised virus control and augmented perforin-mediated immunopathology in IFN-gamma-deficient mice infected with lymphocytic choriomeningitis virus. J. Immunol. 163, 6114–6122 (1999). 10570301

[b60] Becker T. C. . Use of recombinant adenovirus for metabolic engineering of mammalian cells. Methods Cell Biol. 43 Pt A, 161–189 (1994). 7823861 10.1016/s0091-679x(08)60603-2

[b61] Cobbold S. P., Jayasuriya A., Nash A., Prospero T. D. & Waldmann H. Therapy with monoclonal antibodies by elimination of T-cell subsets *in vivo*. Nature 312, 548–551 (1984). 6150440 10.1038/312548a0

[b62] Qin S., Cobbold S., Tighe H., Benjamin R. & Waldmann H. CD4 monoclonal antibody pairs for immunosuppression and tolerance induction. Eur. J. Immunol. 17, 1159–1165 (1987). 2441998 10.1002/eji.1830170813

[b63] Steffensen M. A. . Qualitative and quantitative analysis of adenovirus type 5 vector-induced memory CD8 T cells: not as bad as their reputation. J. Virol. 87, 6283–6295 (2013). 23536658 10.1128/JVI.00465-13 PMC3648097

