# Supplementary material for: Combined local and systemic immunization is essential for durable T-cell mediated heterosubtypic immunity against influenza A virus
Source: Sci Rep. 2016 Feb 1;6:20137. doi: 10.1038/srep20137 (PMC4735591; doi:10.1038/srep20137)
Supplement: Supplementary Information [file srep20137-s1.pdf]

Combined local and systemic immunization is essential for durable T-cell mediated heterosubtypic immunity against influenza A virus

Ida E M Uddback[#], Line M I Pedersen[#], Sara R Pedersen, Maria A Steffensen, Peter J Holst, Allan R Thomsen, Jan P Christensen*

Department of Immunology and Microbiology,

University of Copenhagen,

Copenhagen, Denmark

[#]These authors contributed equally to the paper

Correspondence and requests for materials should be addressed to Jan Pravsgaard Christensen, Department of Immunology and Microbiology, University of Copenhagen, The Panum Institute, Building 22.5.16, 3C Blegdamsvej, DK-2200 Copenhagen N, Denmark. E-mail: jpc@sund.ku.dk

Supple. Figure 1: Gating strategy for flow cytometric analysis. All events were first depicted using a combination of forward scatter area (FSC-A) and side scatter area (SSC-A). Lymphocytes were gated and displayed using a combination of FSC-A and forward scatter-height (FSC-H), singlets were gated. Depending on whether we included the use of a dump channel or not, cells were displayed using either a combination of anti-B220 or anti-CD8 or anti-CD44 and anti-CD8. In both cases CD8 T cells were selected, and the fraction of cytokine positive cells was determined. It should be stressed that similar results were obtained independent of staining and gating strategy.

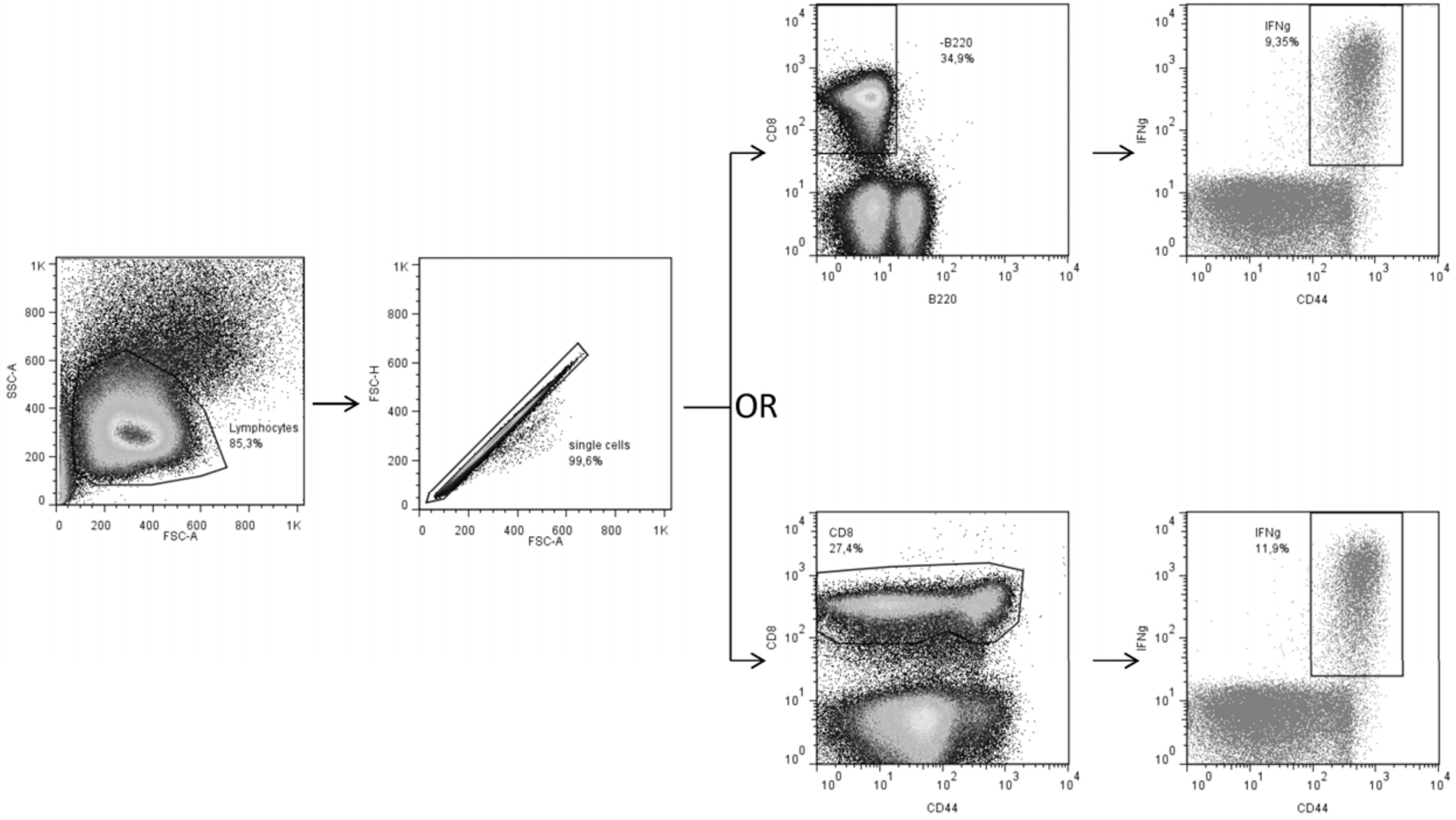

Lymphocytes
85,3%
SSC-A
FSC-A
single cells
99,6%
FSC-H
FSC-A
OR
-B220
34,9%
CD8
B220
IFNg
9,35%
IFNg
CD44
CD8
27,4%
CD8
CD44
IFNg
11,9%
IFNg
CD44
